# Activity Predicts Male Reproductive Success in a Polygynous Lizard

**DOI:** 10.1371/journal.pone.0038856

**Published:** 2012-07-11

**Authors:** J. Scott Keogh, Daniel W. A. Noble, Eleanor E. Wilson, Martin J. Whiting

**Affiliations:** 1 Division of Evolution, Ecology and Genetics, Research School of Biology, The Australian National University, Canberra, Australia; 2 Department of Biological Sciences, Division of Brain, Behaviour and Evolution, Macquarie University, Sydney, Australia; University of Arizona, United States of America

## Abstract

Activity patterns and social interactions play a key role in determining reproductive success, although this is poorly understood for species that lack overt social behaviour. We used genetic paternity analysis to quantify both multiple paternity and the relative roles of activity and social behaviour in determining reproductive success in a nondescript Australian lizard. During the breeding season we intensively followed and recorded the behaviour of a group of seven males and 13 females in a naturalistic outdoor enclosure to examine the relative roles of body size, activity and social interactions in determining male fertilization success. We found multiple paternity in 42% of clutches. No single behaviour was a significant predictor of male fertilization success in isolation, but male-female association, interactions and courtship explained 41% of the variation in male fertilization success. Males with the highest number of offspring sired invested heavily in interacting with females but spent very little time in interactions with males. These same males also sired offspring from more clutches. When taken collectively, an index of overall male activity, including locomotion and all social interactions, significantly explained 81% of the variation in the total number of offspring sired and 90% of the variation in the number of clutches in which males sired offspring. We suggest that the most successful male strategy is a form of endurance rivalry in which active mate searching and interactions with females have the greatest fitness benefits.

## Introduction

According to sexual selection theory males are predicted to maximize their reproductive success by mating with many females [1]. The success of different males in obtaining mates depends strongly on the resources required by receptive females and their spatial and temporal distribution [2], [3], [4]. In polygynous mating systems, males can achieve high reproductive success by defending resources required by females for mating (resource defence polygyny), defending groups of females (female defence polygyny) or by exhibiting showy, sexual ornaments or displays such as in lekking species. For example, in some species males that display more vigorously at a lek are more likely to be chosen by females [5], whereas in other systems large aggressive males are expected to outcompete rival males for resources or females [1]. Therefore, specific morphological and behavioural traits that enhance a male’s ability to acquire females, and/or the resources that they require, are expected to be targets of selection.

Much of our knowledge about the predictors of male reproductive success come from studies on insects, birds and frogs [1], [6]. In many cases morphological predictors such as male body size or armaments are correlated with high reproductive success and are most common in mating systems where males monopolise resources used by females or females themselves [7]. As a consequence, many studies target species in which males are elaborately ornamented or show clear sexual dimorphism [1]. We know much less about species lacking clear sexual dimorphism and which may use less obvious tactics to secure paternities. Furthermore, in mating systems where females are dispersed, are sexually receptive for short periods, and where resources are less important for them, male behavioural attributes may be more important contributors to reproductive success [8], [9], [10], [11]. In such situations, selection for behavioural attributes that allow males to persist at a breeding site for long periods of time (endurance rivalry) or that promote increased interactions with females when they are receptive, are predicted to be under selection.

The Southern Water Skink (*Eulamprus heatwolei*) is distributed widely across southern Australia, is viviparous, and females give birth to 1–5 offspring per litter. The mating system of *E. heatwolei* is highly polgynous. Many adult females establish home ranges close to river edges where there is an abundance of large logs and fallen debris and the home ranges of males overlap an average of 2.29 females [12]. However, both males and females exhibit alternative reproductive tactics (territorial or floater) that form part of a behavioural syndrome [12], [13], [14]. In novel environments in the lab, floater males are more active then territorial males and spend more time feeding [13]. In an anti-predator context, floater males are more likely to flee into a refuge and have a longer latency to emerge [13]. In the wild, larger territorial males were more likely to father an entire clutch or share paternity with fewer other sires than smaller territorial males but floater males tended to father heavier offspring [14]. However, both small and large floater and territorial males sire offspring with neither strategy clearly advantageous over the other, and many males sire no offspring [12], [14]. The Southern Water Skink (*Eulamprus heatwolei*) is a good species in which to test the relative importance of behaviour in sexual selection because females display high levels of multiple paternity [12], [14], there is high variation in male reproductive success [12], [14], and males and females have different behavioural phenotypes [14]. It was not possible in our previous studies on a wild population to assemble highly detailed information on social interactions and activity levels relative to mating success. Here we combined intensive behavioural observation of an adult group of lizards in a single large naturalistic enclosure with genetic paternity data to test the hypothesis that the proportion of time a male is observed in active behaviours predicts male fertilization success.

## Results

We established a breeding population of 13 female and seven male lizards in a single large semi-natural enclosure (10×10 m) where we could conduct detailed behavioural observations on each of the lizards during the breeding period. All 13 females from our large outdoor enclosure were collected at the end of the breeding period. Twelve of the thirteen females gave birth to a total of 37 offspring. Litter size ranged from one to four offspring (mean = 3.1, SE = 0.23). Paternity was assigned with 100% certainty to all offspring. Seven out of 12 litters had one father and multiple paternity was identified in the remaining five (42% of clutches). Of these, four had two fathers and one had three fathers in a litter of three offspring. Male reproductive success was not significantly related to male body size (Table 1). A small male sired the most offspring: 14 out of 37. Apart from this small male, there was a trend for large males to sire more offspring than small males. Only one small male failed to sire any offspring in this experiment. Seven copulations were observed and four of these resulted in offspring.

**Table 1:** Regression analyses of the active behaviour categories, all active behaviours combined and male body size, relative to the total number of offspring sired.

Behaviour	R^2^	F_1,7_	P
General activity	0.09	0.471	0.5229
Interactions with females	0.41	3.484	0.1209
Interactions with males	−0.17	1.000	0.3632
All active behaviours	0.81	21.202	0.0154
Snout-vent length	0.09	0.515	0.5054

**Figure 1:**
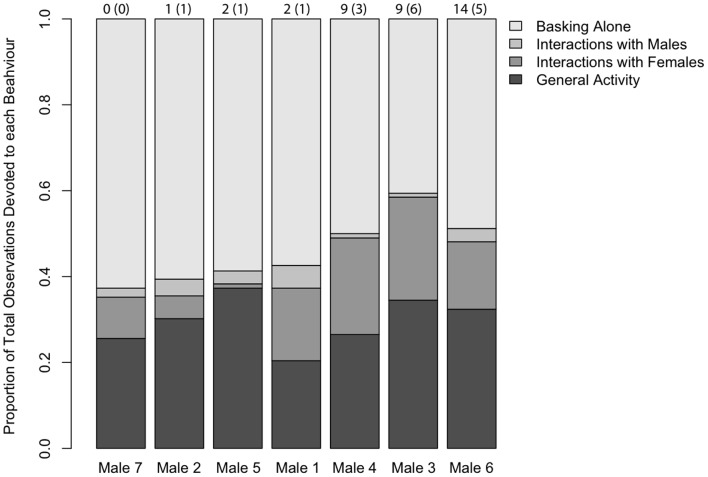
Summary of the proportion of activity for each behavioural category for each male. Total number of offspring sired by each male is shown above each bar with the number of clutches in parentheses.

**Figure 2:**
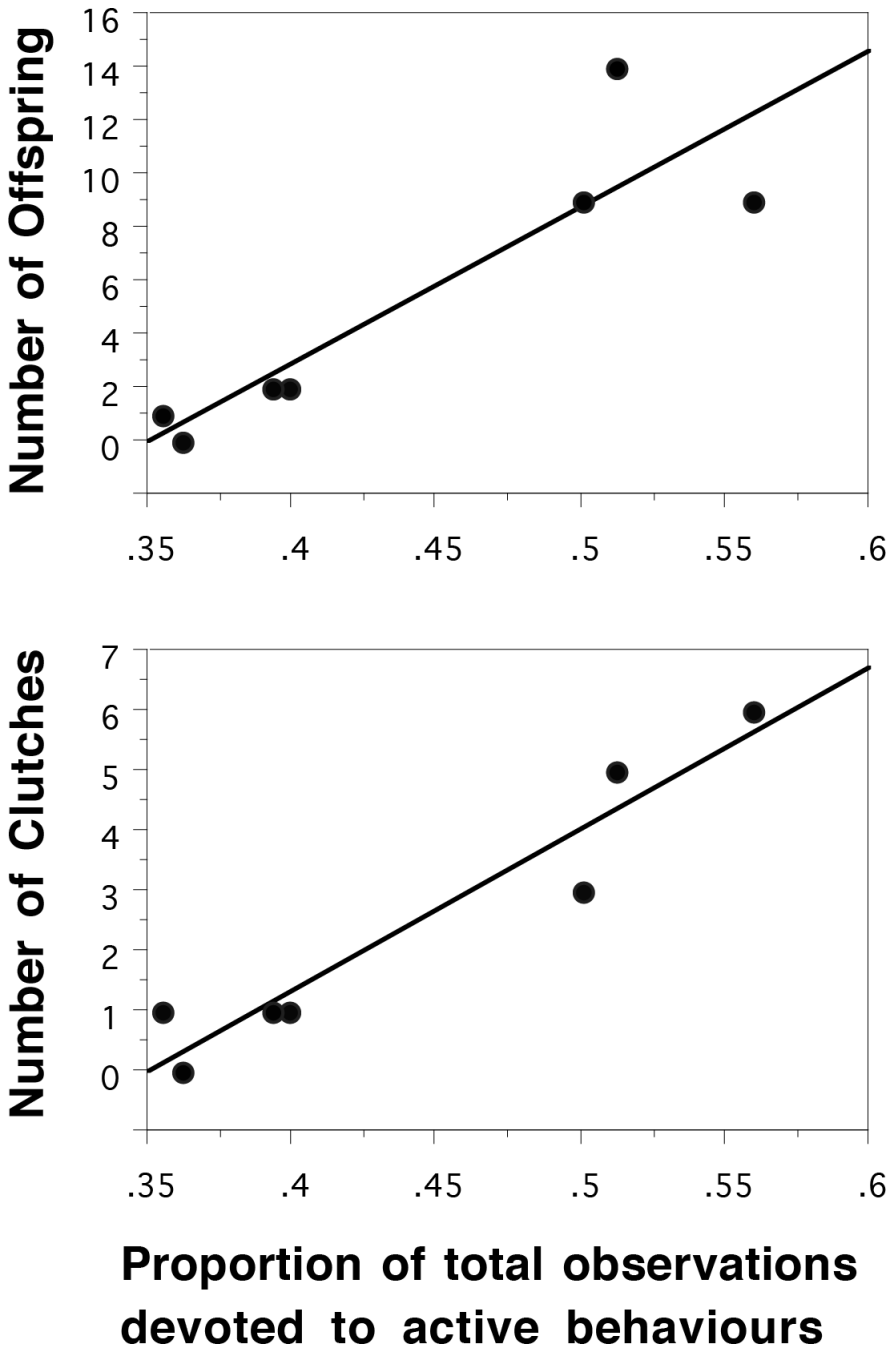
The relationship between active behaviours with other lizards relative to the number of offspring sired (top) and the number of clutches in which males sired offspring (bottom).

**Figure 3:**
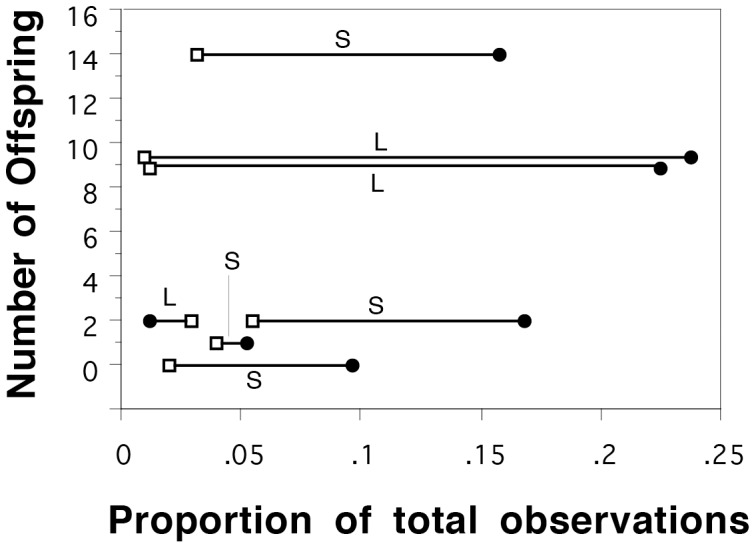
The proportion of behaviour related to aggressive interactions with other males (open squares) relative to active interactions with females (filled circles) for each male as a function of the number of offspring sired. Male body size is noted where “S” refers to small males (88–89 mm) and “L” refers to large males (95–96 mm). Males that spent a greater proportion of time interacting with females also tended to spend comparatively little time interacting with rival males.

During the breeding season we recorded 1119 behavioural observations for the 7 males in our enclosure (range, 115–261 observations) that could be divided into four behavioural categories (Figure 1). All categories of behaviour were examined as possible predictors of male fertilization success but none of them individually were significant predictors (Table 1). However, interactions with females explained 41% of the variation in male fertilization success (Table 1). When all active male behaviours were taken into account, including both social interactions and general locomotion, there was a strong and significant positive correlation that explained 81% of the variation in male fertilization success (Table 1; Figure 2). Males that exhibited more active behaviours also sired offspring from more clutches (Figure 2; R^2^ = 0.9; F _1,7_ = 56.12; P = 0.002). Conversely, there was a negative relationship between the proportion of behaviours males devoted to interacting aggressively with other males and male fertilization success (Table 1). Therefore, males that had the highest fertilization success invested heavily in interacting with females but comparatively little in interactions with males (Figure 3).

## Discussion

We have quantified in a very direct way the activity level of individual males, how they divide total activity between aggressive interactions with other males and interactions with females, and how that activity relates to fitness. We show that males exhibiting a greater proportion of active behaviours during the breeding season sired more offspring in a greater number of clutches, irrespective of male body size.

The correlation between reproductive success and activity could be explained by two subtly different (and not necessarily exclusive) processes. First, more active males may increase their reproductive success by remaining active over a greater number of days increasing the number of receptive females they interact and copulate with (endurance rivalry). Attendance at breeding sites has been shown to be an important determinant of reproductive success in many vertebrate species. Salvador *et al.* [10] followed a population of Cyren’s Rock Lizard (*Iberolacerta cyreni*) for two consecutive years and found that activity level (number of times observed during the breeding season) strongly predicted male reproductive success. More active males gained access to mates by having more females within their home range [10]. In the Galapagos sea lion (*Zalophus wollebaeki*) male reproductive success was predicted by male attendance at breeding sites and this is due to the long reproductive period (five or more months) in this species [9]. This relationship was also independent of male body size [9] and suggests that body size *per se* may not always be as important in governing male reproductive success as is often assumed. This hypothesis should be particularly prominent in systems where females are temporally variable in their receptivity and the breeding season lasts for a long time [2]. This is unlikely to be the case for *Eulamprus heatwolei* because females are known to have a relatively short receptive period of only one to two weeks during mid October [15]. Such a restrictive receptive period suggests that male attendance is unlikely to increase a male’s reproductive success although copulations before the breeding period and sperm storage may play an important role and this hypothesis cannot be ruled out.

The alternative hypothesis is that more active males are likely to traverse their environment and increase their probability of encountering receptive females, providing males with a greater number of mating opportunities. Male Common Lizards (*Lacerta agilis*) with elevated testosterone levels have been shown to move greater distances then control males and mate with more females [16]. In the North American Red Squirrel (*Tamiasciurus hudsonicus*) male search effort (home range area) and search ability (number of receptive females encountered during the mating season) both correlated with the number of matings a male obtained and his actual reproductive success [8]. Our data provides support for the latter hypothesis as males that gained more offspring interacted with more females and sired offspring from more clutches. This is also consistent with the short and near simultaneous female receptive period in the wild [15]. Finally, males clustered into two groups: four males with low activity and low numbers of offspring sired and three males with high levels of activity and higher numbers of offspring sired. A larger sample size is needed to test whether this bimodal distribution correlates with discreet alternate reproductive tactics, but this is entirely possible given that previous work on this species has documented floater and resident males in the same population [12], [14].

In our study 42% of litters had more than one sire. Paternity analysis of offspring born from wild collected females from the same population showed the proportion of multiple paternity to range from 57–64.7% [12], [14], which suggests that many females are promiscuous. There are a number of adaptive hypotheses as to why females mate multiply. First, females may benefit directly by ensuring against sperm limitation [17], [18]. This can result from a high rate of infertility or sperm deformations among males or due to the inadequate transfer of sperm during copulation [17]. Alternatively, females may gain indirect fitness benefits by mating with more than one male if they gain ‘good genes’ through the promotion of sperm competition or disease resistance [19], [20] or by increasing the probability of being fertilized by a male that is genetically compatible with herself [21]. However, multiple mating may not always be adaptive to females and their tendency to do so could be a result of direct selection on male mating behaviour followed by correlated indirect selection on female mating rates [22]. Females are able to easily reject males [15] but are also promiscuous [12]. The benefits of multiple mating for females in this species are as yet unclear, but in other lizard species such as the Sand Lizard (*Lacerta agilis*), mating with multiple males results in higher offspring viability [23]. Furthermore, reproductive success in *L. agilis* is tied to mate searching and encounter rates. During warmer periods male *L. agilis* are able to spend more time actively searching for females and thereby potentially increasing rates of multiple paternity [24]. Similarly, the high reproductive success (both in total numbers and number of clutches) of more active male *E. heatwolei* suggests that selection on male activity levels is strong.

In conclusion, our results demonstrate the importance of activity related behaviours in sexual selection in a species that lacks visual ornamentation and which has low display rates. Dynamic behavioural traits may play a more important role in intrasexual competition than previously thought in reptiles and future work quantifying the relative roles of different behavioural traits to male reproductive success promises to be a fruitful area of study.

## Methods

### Study Animal

We collected adult *E. heatwolei* from 24 September until 5 October, immediately after spring emergence, from a large population in the Tidbinbilla Nature Reserve, 25 km southwest of Canberra in the Australian Capital Territory (elevation 800 m). We measured snout-vent length (SVL) and tail length to the nearest mm, head length and head width to the nearest 0.1 mm, and weight to the nearest 0.1 g. Individuals were sexed by checking for hemipenes. Lizards were marked individually with a unique toe-clip combination and the toes and 5 mm of tail tip were retained for genotyping. Natural toe loss is common in *E. heatwolei* (JSK pers. obs.) and toe-clipping has been shown to have no effect on lizard behaviour or fitness in a closely related species [25]. All individuals used in this study had complete or fully regrown tails, were free of visible parasites, and appeared to be in good health at the onset of the experiment.

### Experimental Enclosure and Lizard Husbandry

We used a single large (10×10 m) enclosure with high quality habitat (an abundance of logs and refugia) where we recorded detailed data on all social interactions and other behaviours of males to compare with paternity data. The large size of the enclosure allowed natural home-range establishment and behaviour but it was small enough to allow us to observe the entire enclosure at once. The density of lizards in the enclosure approximates natural densities in high quality habitat during the mating season (Morrison et al. 2002).

Thirteen adult females (96–104 mm SVL) and seven adult males (88–96 mm SVL) were introduced into the enclosure on 6 October. For ease of identification, we painted a unique number on the dorsum of each lizard using non-toxic, xylene-free paint pens. We observed the enclosure from 7 October - 21 November, including two weeks after the mating season ended and lizard behaviour had obviously decreased.

Lizards in the enclosures always had access to water and fed on insects that naturally occurred in the enclosures. We supplemented their diet with wet dog food twice per week. Females were brought into the laboratory on 8 January to give birth and housed in individual snap-lock containers (30L×21W×9H cm), in a temperature-controlled environment (18°C) with a natural light cycle (12 h light: dark). The lizards were provided with bark chip bedding, a cardboard retreat site, and heat tape for basking (30°C) eight hours a day to allow natural thermoregulation. The lizards were provided with fresh water *ad libitum* and dog food and mealworms every second day. The females were checked twice daily until they gave birth. Neonates were removed upon discovery and housed separately.

### Behavioural Sampling

During observation periods one person (EEW) walked around the perimeter of the enclosure and continuously scanned for all activity related behaviours and social interactions. Observations were recorded from 1000–1600 h when lizards are most active and only on days when conditions favoured lizard activity (warm, sunny, calm). In order to facilitate recording observations on multiple lizards at once, we broadly divided activity into four basic categories, but because we had a manageable number of lizards, it was rarely the case that multiple interactions were happening at the exact same time. The first category was general activity related behaviours with no obvious receiver. These behaviours involved locomotion (movement of an individual greater than 2 cm from its initial position) and head-bobbing (stereotypical up and down movement of the head and neck) where no conspecific was observed. We also recorded interactions between two individuals. Interactions involving a male and female included courtship, chasing (when a male actively chased a female), copulations or when a male was observed within 5 cm of the female while basking (stationary in the sun) or being stationary (stationary in the shade). Interactions involving two males included fighting (biting) and chasing when one individual actively displaced the other by chasing him through the enclosure. In each case we recorded the type of behaviour, the individuals involved, and the onset time of interactions. Because behaviours were performed rapidly and generally lasted only a few seconds, we recorded occurrence instead of duration. If multiple behaviours were displayed in rapid succession by a single individual, we scored only the most dominant behaviour exhibited (for example, courtship or fighting over chasing).

### Paternity Assignment

Neonates were individually toe-clipped and approximately 5 mm of tail tip was removed for genotyping. All offspring, mothers and potential sires were genotyped for three highly polymorphic microsatellite loci, Ek37, Ek100, Ek107, as described in Scott *et al.* [26] and Morrison *et al*. [12]. We assigned paternity to offspring manually by first matching maternal alleles in the offspring and then going through the alleles of all the potential fathers until there was a match at all three paternal alleles.

### Data Analyses

We divided the 6.5 week observation period into three time periods: acclimatisation, the mating period, and post-mating period. The mating period is relatively short and we demonstrated in an earlier study that social interactions during this period are very different than before and after the mating period [15]. Therefore, we excluded all of the behavioural data before and after the mating period and focused our analyses on the 19-day mating period only (October 20 - November 8). We calculated the total number of behavioural events for each male and then expressed each behavioural category as a proportion of his total, thereby controlling for unequal sampling duration. We performed a series of linear regressions between the total number of offspring sired and the proportion of the total number of observations invested in each category of behaviour. We also examined these behaviours in the context of male body size (snout-vent length presented by results similar if mass was used instead) and the number of clutches in which a male sired offspring. For each male we also examined the difference between the relative number of social interactions with females and aggressive interactions with males.

### Ethics Statement

All work carried out as part of this project was done under the approval of the Australian National University Animal Experimentation Ethics Committee (F.BTZ.01.99) and with research permits from Environment ACT (permit number LT1999008). Tidbinbilla Nature Reserve gave us permission to capture the animals used in this study.
